# Altered endometrial oestrogen-responsiveness and recurrent reproductive failure

**DOI:** 10.1530/RAF-21-0093

**Published:** 2022-02-21

**Authors:** Hannan Al-Lamee, Amy Ellison, Josephine Drury, Christopher J Hill, Andrew J Drakeley, Dharani K Hapangama, Nicola Tempest

**Affiliations:** 1 Centre for Women’s Health Research, Department of Women’s and Children’s Health, Institute of Life Course and Medical Sciences, University of Liverpool, Member of Liverpool Health Partners, Liverpool, UK; 2 Liverpool Women’s NHS Foundation Trust, Member of Liverpool Health Partners, Liverpool, UK; 3 Hewitt Centre for Reproductive Medicine, Liverpool Women’s NHS Foundation Trust, Liverpool, UK

**Keywords:** oestrogen-receptor beta, recurrent reproductive failure, recurrent pregnancy loss, recurrent implantation failure, endometrium, window of implantation

## Abstract

**Graphical abstract:**

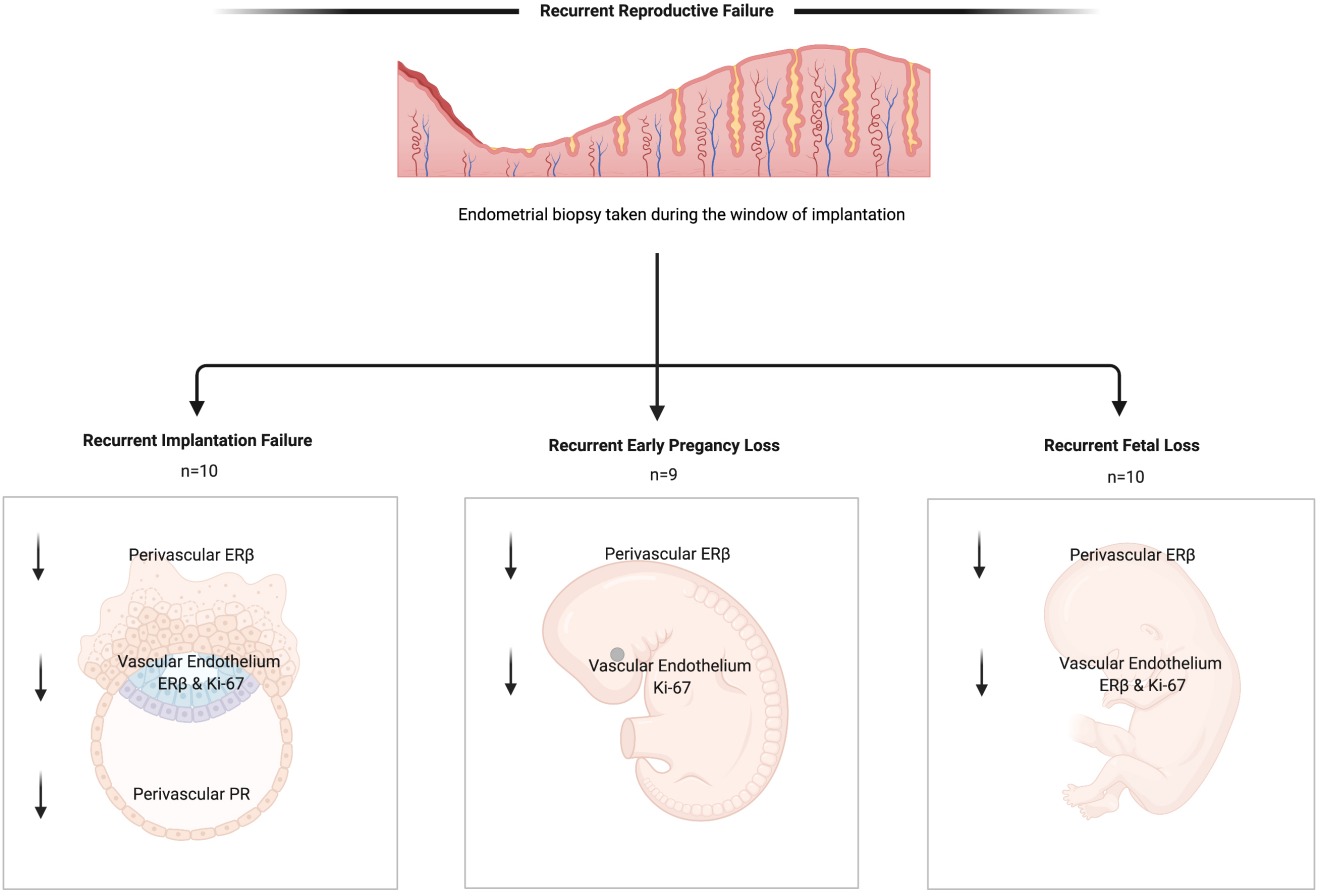

**Abstract:**

Recurrent reproductive failure (RRF) encompasses recurrent implantation failure (RIF) and recurrent pregnancy loss (RPL). These highly prevalent, distressing conditions have many unanswered questions regarding aetiology and management. Oestrogen receptor beta (ERβ) is the predominant oestrogen receptor expressed in the vascular endothelium of the endometrium during the window of implantation (WOI). The establishment of normal endometrial receptivity is integrally associated with progesterone receptor (PR). Therefore, we aimed to investigate whether women with RRF have clinical, type-specific endometrial aberrations of ERβ, PR and Ki-67 expression during the WOI. Thirty-eight endometrial biopsies were collected; 29 RRF (10 RIF, 9 recurrent loss of early pregnancy (RLEP) and 10 recurrent fetal loss (RFL)) and 9 fertile controls (FC). Within RIF, RLEP and RFL groups, the perivascular compartment showed significantly lower levels of ERβ vs FC (*P  = *0.02*, P  = *0.03 and *P  = *0.01, respectively). Vascular endothelium also displayed significantly lower levels of ERβ within RIF and RFL cohorts vs FC (*P  = *0.03 and *P  = *0.003). The expression of Ki-67 was significantly lower within vascular endothelium of all RRF; RIF (*P  = *0.02), RLEP (*P  = *0.02) and RFL (*P  <*0.01). PR was significantly reduced (*P  <*0.001) in the perivascular area of women with RIF. These findings provide novel insights into biological correlates of clinical subtypes of RRF. The endometrium of women with RRF display significantly altered levels of ERβ, PR and Ki-67 during the WOI, furthering our understanding of the defective endometrial phenotype of women suffering from RRF, with possible impaired glandular function, angiogenesis and decidualisation.

**Lay summary:**

Recurrent reproductive failure (RRF) refers to a group of devastating conditions with many unanswered questions regarding their causes and treatment options. The lining of the womb, the endometrium, is primed and suitable for successful embryo implantation for a short time during the menstrual cycle; the window of implantation (WOI). Oestrogen is a key hormone that plays an important role in regulating the endometrium and its effects are exerted via two oestrogen receptor subtypes. Oestrogen receptor beta (ERβ) is the main oestrogen receptor present during the WOI. Progesterone receptor allows the other main hormone, progesterone, to influence the endometrial activity and Ki-67 reflects the proliferative activity of the cells within the endometrium. We investigated the expression of these markers in endometrial samples collected from women with RRF and proven fertility. We found that the endometrium of women with RRF has significantly lower levels of ERβ and Ki-67 during the WOI, possibly leading to unsuccessful pregnancies.

## Introduction

Recurrent reproductive failure (RRF) refers to a group of devastating conditions with many unanswered questions. RRF can be subcategorised into one of the following manifestations: (i) recurrent implantation failure (RIF); (ii) recurrent pregnancy loss (RPL) (Polanski *et al.* 2014, Kolte *et al.* 2015, Cimadomo *et al.* 2021) and both conditions are inconsistently defined. Pregnancy loss, defined as the spontaneous loss of a pregnancy prior to fetal viability, is extremely common, estimated to occur in 15–20% of all clinical pregnancies (Morley *et al.* 2013, Jia *et al.* 2015, ESHRE Guideline Group on RPL *et al.* 2018).

RIF is most commonly accepted as the unsuccessful transfer of three or more good quality embryos following *in vitro*fertilisation (IVF) and affects approximately 10% of women undergoing IVF treatment (Margalioth *et al.* 2006, Tempest *et al.* 2021). Inadequate endometrial receptivity is said to be responsible for two-thirds of RIF (Simon & Laufer 2012).

RPL affects 1–2% of all couples (ESHRE Guideline Group on RPL *et al.* 2018) and is defined in most studies as the loss of two (Practice Committee of the American Society for Reproductive Medicine 2013, ESHRE Guideline Group on RPL *et al.* 2018) or three consecutive pregnancies (RCOG Green-top Guideline No. 17 2011). RPL can be subcategorised into recurrent loss of an early pregnancy (RLEP) (prior to 10 weeks of gestation) and recurrent fetal loss (RFL) (post 10 weeks of gestation) (Kolte *et al.* 2015). RPL can be described as an under-selective state of the endometrium with an inappropriately high receptivity (Larsen *et al.* 2013, Macklon & Brosens 2014).

RRF is one of the most difficult challenges for clinicians in reproductive medicine, with approximately 50% of sufferers left with no explanatory cause for the condition, despite extensive investigation (El Hachem *et al.* 2017) resulting in significant emotional and psychological impact for the women and their partners (Farren *et al.* 2020). Many studies have suggested an association between unexplained RRF and a defective endometrium (Quenby *et al.* 2007, Ticconi *et al.* 2019). The human endometrium is receptive to a blastocyst for a brief and defined period of time termed the window of implantation (WOI) (Diedrich *et al.* 2007, Wang *et al.* 2020), meaning a receptive endometrial environment is an essential prerequisite for successful embryo implantation and development (Wang & Dey 2006, Kelleher *et al.* 2018, Wang *et al.* 2020).

Oestrogenic effects on the endometrium are exerted via two classical oestrogen receptor (ER) isoforms, oestrogen receptor-α (ERα) and oestrogen receptor β (ERβ). Although these receptors are highly homologous, they elicit unique cellular effects and exhibit distinctive spatial and temporal endometrial distribution (Hapangama *et al.* 2015). During the menstrual cycle, well-established oestrogen-dependent endometrial cellular proliferation during the follicular phase is primarily mediated by ERα, while ERβ and progesterone receptor (PR) are widely reported to have opposing anti-proliferative effects (Kamal *et al.* 2016). ERβ is thought to prevent undesired ERα-mediated actions of oestradiol (E2) (Hapangama *et al.* 2015) and is the dominant ER subtype expressed within the vascular endothelium during the WOI (Critchley *et al.* 2001). It is therefore believed to be important in angiogenesis and vascular remodelling, both of which are essential for implantation and maintenance of pregnancy (Critchley *et al.* 2001). Along with PR, ERβ is a key regulator of endometrial decidualisation; ERβ knock-out mice display a subfertile phenotype, with distorted luminal epithelial (LE) cells, loss of epithelial differentiation, exaggerated endometrial epithelial proliferation and upregulation of PR (Wada-Hiraike *et al.* 2006, Hapangama *et al.* 2015).

As both ERβ and PR are seen to be important regulators expressed during the WOI, we hypothesised that aberrant expression of these steroid hormone receptors and Ki-67 (a protein marker associated with cellular proliferation) may be seen across different endometrial cellular subtypes of women with RRF.

## Materials and methods

### Tissue samples

Collection and use of all samples described in this manuscript were approved by the Liverpool Adult Ethics committee (REC references; 05/Q1505/115 and 05/Q1505/014). Informed written consent was obtained from all participants. Endometrial biopsies were collected from 38 women: 29 RRF (10 RIF, 9 RLEP and 10 RFL) and 9 controls with proven fertility (≥2 healthy pregnancies) in the WOI (urinary luteinising hormone + 7 ± 2 days). All women included had regular menstrual cycles, no known endometrial pathology or use of contraceptive hormonal medications for 3 months prior to sampling at Liverpool Women’s NHS Foundation Trust. Women were included in the RIF cohort after ≥4 embryo transfers and no positive pregnancy test, RLEP cohort after ≥3 consecutive losses of an early pregnancy or RFL cohort after ≥3 fetal losses. Women with RRF were fully screened for any possible contributing factors and were excluded if screened positive for antiphospholipid syndrome (lupus anticoagulant, immunoglobulin G/M and anticardiolipin antibodies), thrombophilia (activated protein C resistance, Leiden factor V mutation, prothrombin gene mutation, protein C and S deficiency and antithrombin III deficiency), uterine anomaly (as seen on transvaginal ultrasonography), polycystic ovarian syndrome (diagnosed using Rotterdam criteria) (Wang & Mol 2017), diabetes (fasting blood glucose), thyroid function test or parental balanced translocations (peripheral blood karyotyping).

### Immunohistochemistry

Immunohistochemistry was performed on 4-µm paraffin-embedded sections as previously described (Valentijn *et al.* 2013, Maclean *et al.* 2020, Alnafakh *et al.* 2021). Sections were dried, dewaxed and rehydrated prior to heat-induced antigen retrieval in a pressure cooker containing 0.01 M citrate, pH 6.0 for 2 min. Endogenous peroxidase activity was blocked with 0.3% hydrogen peroxide (Sigma Aldrich) for 10 min before applying 2.5% Normal Horse Serum (Vector Laboratories, Oxfordshire, UK) for 20 min at room temperature to block non-specific antigens. Following this, sections were incubated with diluted primary antibody overnight at 4°C (ERβ 1:50 (clone PPG5/10, ab277270; Abcam) and Ki-67 1:200 (clone MM1, MCL-Ki67-MM1-L-CE; Leica Biosystems) or at room temperature for 1 h (PR 1:1000 (clone PgR 636), M3569; Dako). After rinsing, the appropriate secondary antibody was applied for 30 min at room temperature, and visualisation was done with ImmPACT DAB following the manufacturer’s instructions (Vector Laboratories, Peterborough, UK). Sections were counterstained using Gill II Haematoxylin (Thermo Fisher Scientific), dehydrated, cleared and mounted using Consul-Mount (Thermo Fisher Scientific). Matching isotype replaced primary antibody as a negative control, with internal positive control in each staining run. Slides were digitalised using an Aperio CS2 slide scanner (Leica Biosystems).

### Image analysis

A semi-quantitative immunostaining scoring method was used as previously described (Hapangama *et al.* 2008) to quantify ERβ, PR and Ki-67 expression within the endometrial glands, stroma, LE, perivascular and vascular endothelium compartments separately. Staining intensity and distribution were analysed and tissue sections were independently scored by two observers who were blinded to the slides.

### Statistical analysis

Statistical analysis was performed using R software (version 4.0.3), RStudio software (PBC, Boston, MA) and GraphPad-Prism (Version 9). Mann–Whitney *U*test was used to compare groups and statistical significance was inferred at *P  <*0.05 (*), highly significant *P  <*0.01 (**) and very highly significant *P  <*0.001 (***). Box and whisker plots show median (horizontal line), interquartile range (box) and data range (whiskers).

## Results

All women were aged between 27 and 45 years and had a BMI <30 kg/m^2^ (Table 1).

**Table 1:** Participant demographics. Data are presented as median (range) or as* n* (%).

Sample	Control	RIF	RLEP	RFL
*n*	9	10	9	10
Age	36 (30–42)	39 (34–42)	36 (30–42)	34 (27–45)
Live birth	9 (100%)	1 (10%)	5 (56%)	4 (40%)
Failed embryo transfers	0 (0–0)	6 (4–14)	0 (0–0)	0 (0–0)
Early pregnancy loss	0	1 (0–5)	4.5 (3–7)	0 (0–3)
Fetal loss	0 (0–0)	0 (0–0)	1 (0–1)	4 (3–14)

### ERβ expression

**Figure 1:**
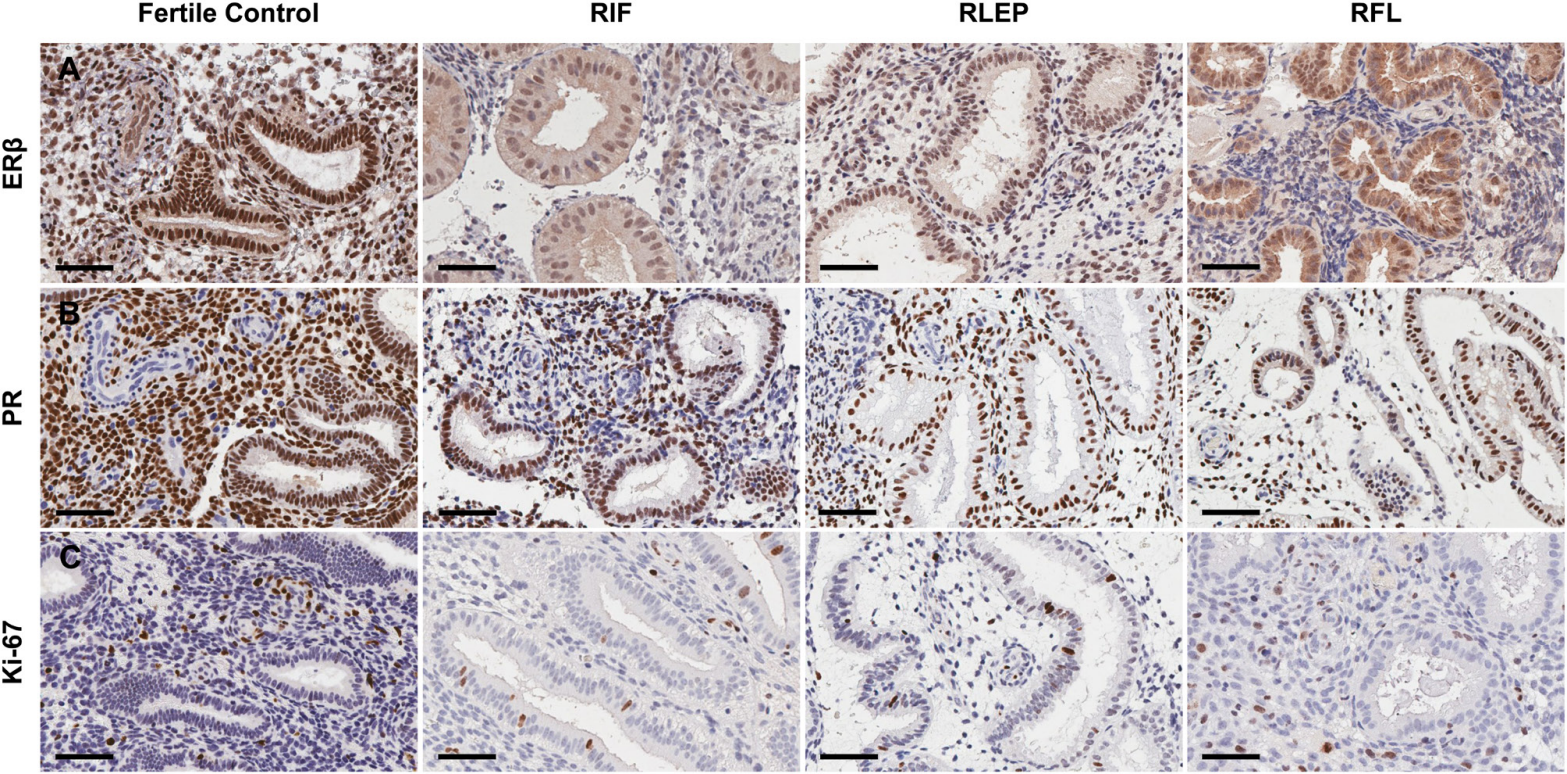
Representative micrographs demonstrating (A) ERβ, (B) PR and (C) Ki-67 immunostaining during the window of implantation in the endometrium of fertile control, recurrent implantation failure (RIF), recurrent loss of early pregnancy (RLEP) and recurrent fetal loss (RFL) patients. Representative images illustrate the distribution of immunostaining across glandular, stromal, perivascular and vascular compartments. Scale bars 60 µm throughout (400× magnification).

**Figure 2:**
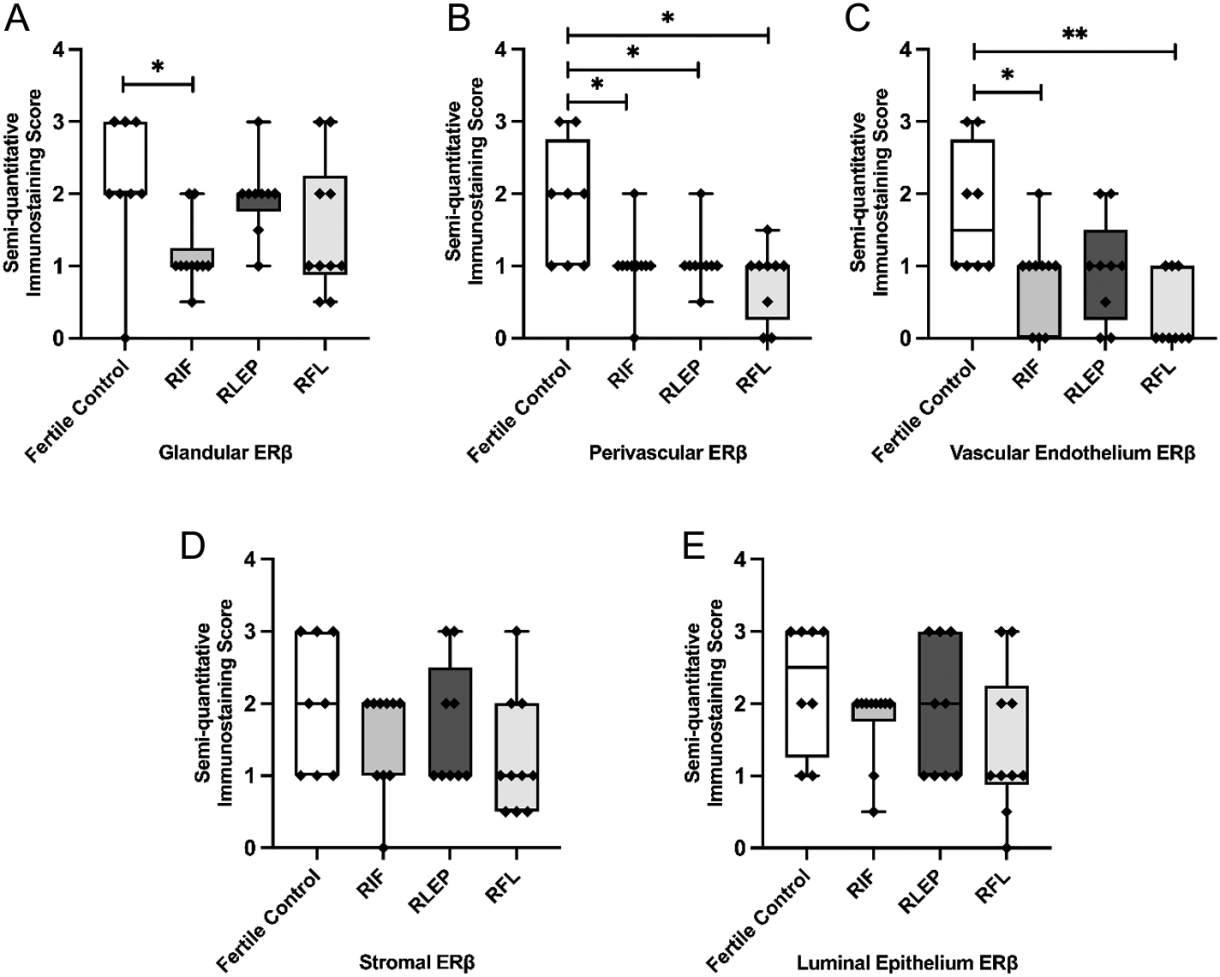
Immunostaining scores for ERβ within the (A) glandular epithelium, (B) perivascular cells, (C) vascular endothelium, (D) stromal cells and (E) luminal epithelium of the endometrium of fertile control, recurrent implantation failure (RIF), recurrent loss of early pregnancy (RLEP) and recurrent fetal loss (RFL) patients.

During the WOI, endometrial glands of women with RIF showed significantly lower levels of ERβ when compared with fertile controls (FC) (*P  = *0.02) (Figs 1A and 2A). The perivascular compartment of all women with RIF, RLEP and RFL showed significantly lower levels of ERβ compared with FC (*P  = *0.02*, P  = *0.03 and *P  = *0.01, respectively) (Figs 1A and 2B). The vascular endothelium also displayed significantly lower levels of ERβ within the RIF and RFL groups compared to FC (*P  = *0.03 and *P  = *0.003, respectively) (Figs 1A and 2C). No significant differences in immunoexpression scores for ERβ were seen within the stromal compartments (Figs 1A and 2D) or LE (Figs 1A and 2E).

### PR expression

**Figure 3:**
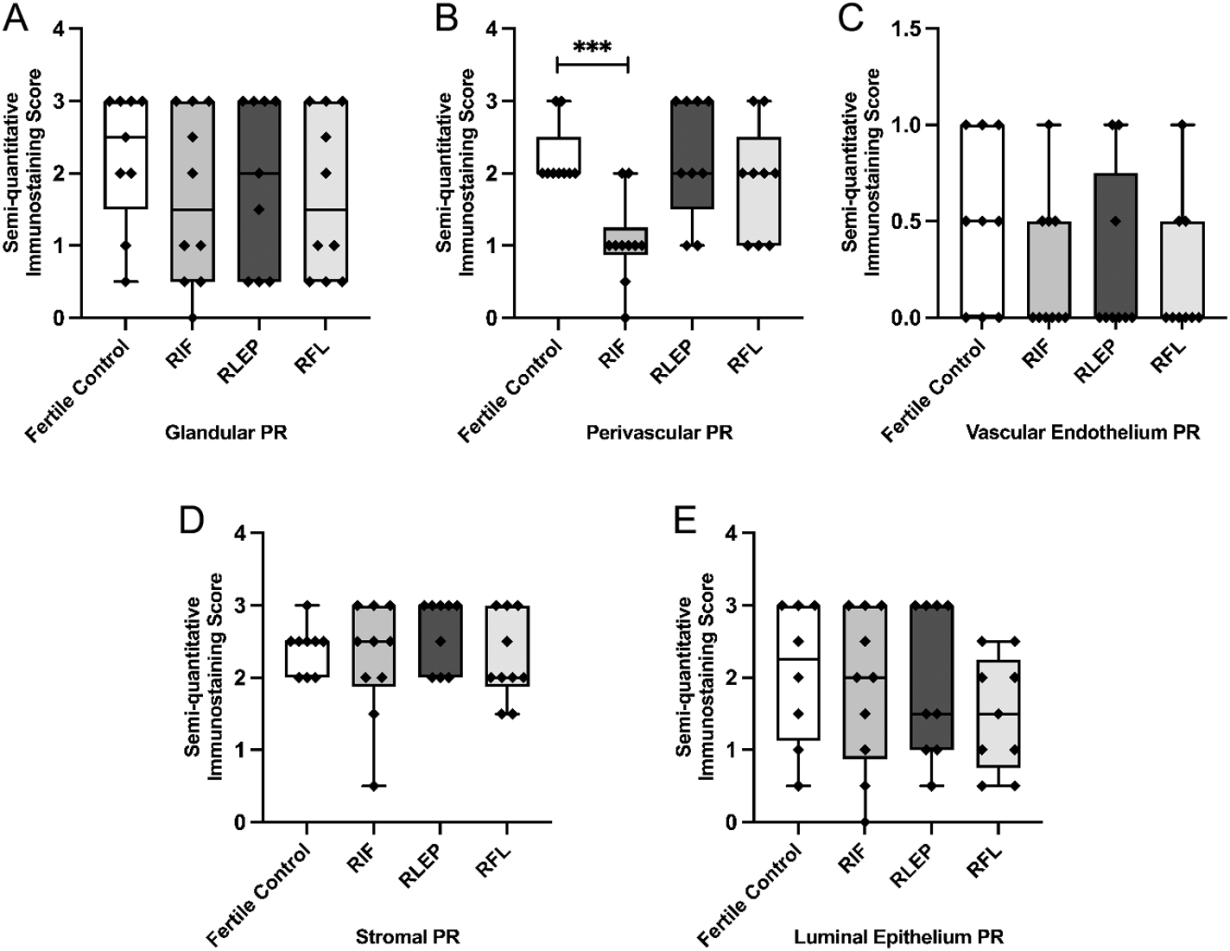
Immunostaining scores for PR within the (A) glandular epithelium, (B) perivascular cells, (C) vascular endothelium, (D) stromal cells and (E) luminal epithelium of the endometrium of fertile control, recurrent implantation failure (RIF), recurrent loss of early pregnancy (RLEP) and recurrent fetal loss (RFL) patients.

PR was found to be significantly reduced (*P  <*0.001) in the perivascular area of women with RIF vs FC (Figs 1B and 3B). No significant differences in immunoexpression scores for PR were seen within the glandular epithelium, vascular endothelial, stromal or LE compartments (Figs 1B and 3A, C, D, E).

### Ki-67 expression

**Figure 4:**
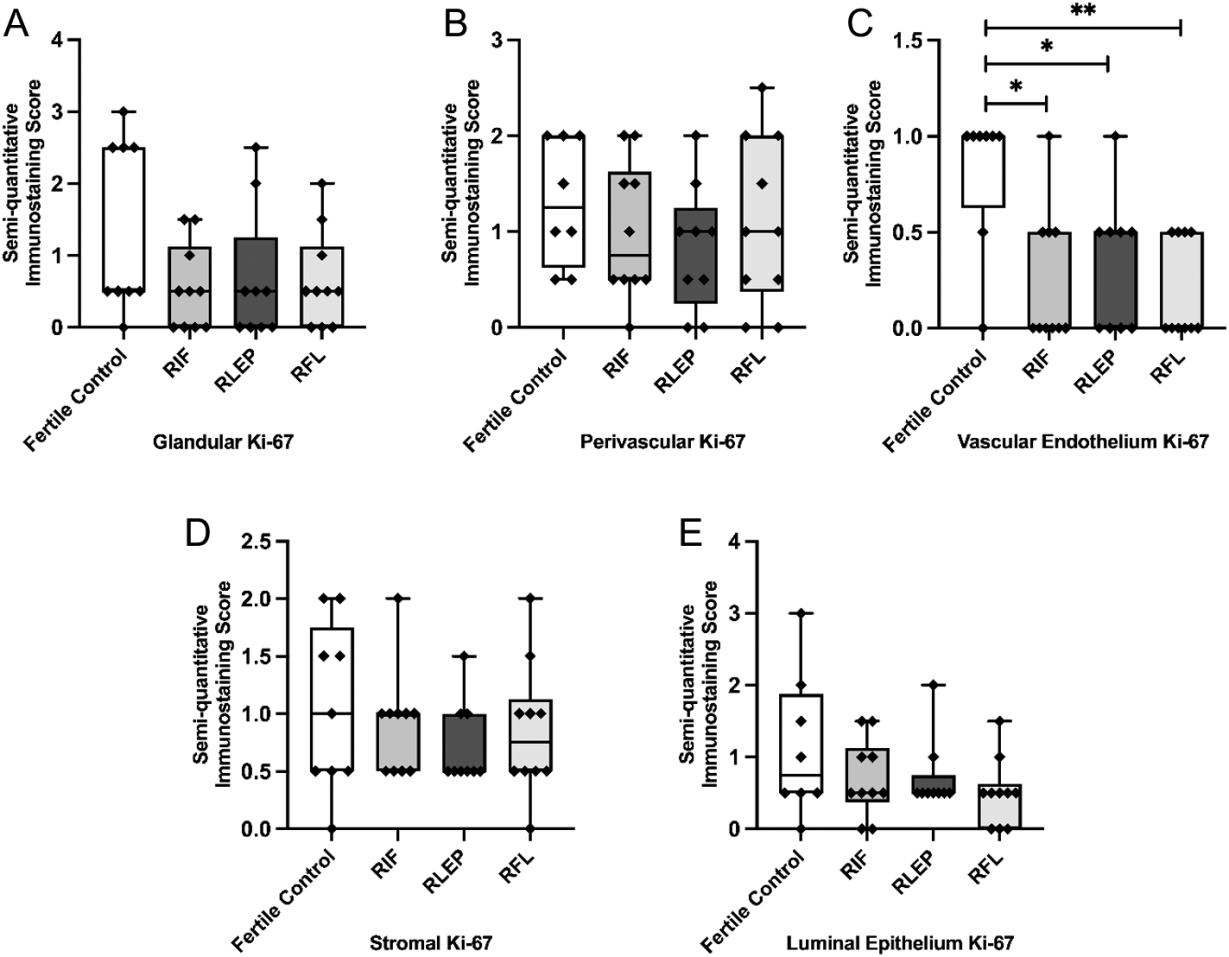
Immunostaining scores for Ki-67 within the (A) glandular epithelium, (B) perivascular cells, (C) vascular endothelium, (D) stromal cells and (E) luminal epithelium of the endometrium of fertile control, recurrent implantation failure (RIF), recurrent loss of early pregnancy (RLEP) and recurrent fetal loss (RFL) patients.

Ki-67 expression was significantly lower within the vascular endothelium of all women with RIF and RPL; RIF (*P  = *0.02), RLEP (*P  = *0.02) and RFL (*P  <*0.01) (Figs 1C and 4C). No significant differences in immunoexpression scores for Ki-67 were seen within the glandular epithelium, perivascular, stromal or LE compartments (Figs 1C and 4A, B, D, E).

## Discussion

Our results demonstrate significantly reduced levels of ERβ, PR and Ki-67 within different endometrial cellular compartments amongst the different clinical subgroups of women with both RIF and RPL when compared to FC.

During the WOI, the endometrial glands of women with RIF show significantly lower levels of ERβ when compared with FC. Uterine glands and their secretions are essential for blastocyst implantation and endometrial decidualisation (Filant & Spencer 2013, Arora *et al.* 2016, Kelleher *et al.* 2018). Previous studies suggest that ERβ plays an important role in the organisation, growth and differentiation of the endometrial epithelium and that a lack of ERβ can lead to abnormal glandular structure and loss of glandular differentiation (Wada-Hiraike *et al.* 2006, Filant & Spencer 2013). Our findings suggest that women with RIF may have impaired glandular function secondary to abnormally low ERβ expression.

Within the RIF group, the perivascular and vascular endothelium also both displayed significantly lower levels of ERβ when compared to FC. Since E2 upregulates the expression of all steroid receptor types, this may suggest a reduced oestrogenic action in the perivascular compartment of the endometrium of women with RIF. Previous studies have shown that ERβ is the predominant form of ER within the vascular endothelium, seen to be markedly increased in vascular cells within the late-secretory period (Critchley *et al.* 2001). Vascular ERβ is expressed during coiling of the spiral arteries and ERβ-positive stromal cells are found within the perivascular region and within decidualised stromal cells, suggesting a key role of this receptor in the modulation of vascular function and during the process of decidualisation (Lecce *et al.* 2001).

Decidualisation and preparation of the endometrium for a successful implantation is controlled by critical target genes downstream of the PR pathway (Wetendorf & DeMayo 2012, Okada *et al.* 2018), and PR was found to be significantly reduced in the perivascular area of women with RIF vs FC. Therefore, reduced perivascular PR expression may be an important feature of a defective endometrial phenotype. Findings from ERβ knock-out mice have shown the continued ability of progesterone to induce a decidual stromal response with high E2 levels, which may also suggest an ERβ-mediated signalling pathway is involved in PR activation (Curtis *et al.* 1999, Lecce *et al.* 2001).

Within the group of women suffering from RPL; the RLEP group showed significantly lower levels of ERβ within the perivascular compartment and the RFL group had significantly lower ERβ within the vascular endothelium compared to FC. Lower ERβ levels within the perivascular and vascular cells of these RPL subgroups may suggest dysfunction in angiogenesis and decidualisation in these women, subsequently leading to pregnancy loss. ERβ has been reported to be expressed in the endometrial endothelial cells throughout the menstrual cycle and is proposed to regulate the angiogenic and vascular changes that occur during implantation and early placentation (Critchley *et al.* 2001). Critchley *et al.* found that treatment with GnRH agonists or ovariectomy (both hypoestrogenic conditions) caused significant reductions in endothelial PR and ERβ expression without altering ERα levels when compared with the late proliferative phase of the normal menstrual cycle (Critchley *et al.* 2001). This further suggests our observation of reduced ERβ and PR in the perivascular region and ERβ and Ki67 in the vascular endothelium of women with RIF and varying RPL subgroups compared with FC may be due to reduced oestrogenic action around the endometrial vasculature of these women. However, this was not correlated with stromal expression of steroid receptors or Ki-67, thus, we can assume the relevant pathway disturbance of this altered perivascular PR and ERβ to be possibly related to the endothelial compartment, where significant alterations were seen.

The presence of Ki-67 was consistently seen to be significantly lower within the vascular endothelium of RIF and both RPL subgroups (RLEP and RFL), suggesting that aberrant or suboptimal vascularisation, resulting in a poor endometrial microenvironment, may be an important aspect of the aetiology of all types of RRF. Further studies should be undertaken to test this hypothesis more robustly and to examine in more detail the differences in abnormal vascularisation between RIF and RPL which may be responsible for their different phenotypes.

This preliminary study provides important insights into the clinical subtype-specific endometrial defects to guide future work. A notable strength of this study is the robust method of screening for the women regarding causes of RRF prior to participation, ensuring the groups were as homongenous as possible. In addition, this work is novel as it analyses the data based on subcategories of RRF and different cellular compartments within the endometrium, which aimed to shed further information about the aetiology of these conditions. Whilst it was designed to be adequately powered to detect statistical significance, generalisation of the results requires confirmation by further studies with a larger sample size. Other limitations of this study include that the endometrial biopsies we utilised sample the functional endometrium only, meaning we did not have access to the basalis (proposed location of endometrial stem cells) tissue. In addition, since this is an observational study without functional data, the possible functional relevance of the observed defects associated with oestrogenic and progestogenic pathways in the RRF endometrium cannot be confirmed.

This data provides novel insights into the biological correlates of clinical types of RRF and suggests that specific alterations in the regulation of endometrial cell fate and oestrogen responsiveness are associated with different types of RRF. Our findings provide a further understanding of the defective endometrial phenotype of women suffering from RRF, with possible impaired glandular function, vascularisation, angiogenesis and decidualisation. The human endometrium contains multiple cell types, each undergoing dramatic transformation throughout the menstrual cycle. Our findings highlight the important requirement to explore the specific areas of the human endometrium according to cellular subtypes, as this may provide clinically useful insights when investigating pathologies such as RRF (Wang *et al.* 2020, Tempest *et al.* 2021). Understanding the aetiology for different clinical subtypes of RRF will enable us to undertake future functional studies and discover novel therapeutic targets.

## Declaration of interest

The authors declare that there is no conflict of interest that could be perceived as prejudicing the impartiality of the research reported.

## Funding

This work was supported by the Wellbeing of Women http://dx.doi.org/10.13039/501100000325 (grant no. RTF510, RG1073 and RG2137). H A L was supported by the Hewitt Fertility Centre/Liverpool Women’s Hospital NHS Trust. A E was supported by the Jean Shanks Foundation http://dx.doi.org/10.13039/501100001308. N T was supported by an Academic Clinical Lectureship from the National Institute of Health Research http://dx.doi.org/10.13039/100005622.

## Author contribution statement

Conceptualisation: H A L, D K H and N T; experiments carried out: A E, J D and H A L; writing and editing: H A L, A E, J D, C J H, H A L, A J D, D K H and N T; figures: H A L, N T, J D and D K H; funding acquisition: A E and D K H. All authors have read and agreed to the published version of the manuscript.
